# PolySearch2: a significantly improved text-mining system for discovering associations between human diseases, genes, drugs, metabolites, toxins and more

**DOI:** 10.1093/nar/gkv383

**Published:** 2015-04-29

**Authors:** Yifeng Liu, Yongjie Liang, David Wishart

**Affiliations:** 1 Department of Computing Science, University of Alberta, Edmonton, T6G 2E8 Canada; 2 Department of Biological Science, University of Alberta, Edmonton, T6G 2E6 Canada

## Abstract

PolySearch2 (http://polysearch.ca) is an online text-mining system for identifying relationships between biomedical entities such as human diseases, genes, SNPs, proteins, drugs, metabolites, toxins, metabolic pathways, organs, tissues, subcellular organelles, positive health effects, negative health effects, drug actions, Gene Ontology terms, MeSH terms, ICD-10 medical codes, biological taxonomies and chemical taxonomies. PolySearch2 supports a generalized ‘Given X, find all associated Ys’ query, where X and Y can be selected from the aforementioned biomedical entities. An example query might be: ‘Find all diseases associated with Bisphenol A’. To find its answers, PolySearch2 searches for associations against comprehensive collections of free-text collections, including local versions of MEDLINE abstracts, PubMed Central full-text articles, Wikipedia full-text articles and US Patent application abstracts. PolySearch2 also searches 14 widely used, text-rich biological databases such as UniProt, DrugBank and Human Metabolome Database to improve its accuracy and coverage. PolySearch2 maintains an extensive thesaurus of biological terms and exploits the latest search engine technology to rapidly retrieve relevant articles and databases records. PolySearch2 also generates, ranks and annotates associative candidates and present results with relevancy statistics and highlighted key sentences to facilitate user interpretation.

## INTRODUCTION

Keeping pace with the rapidly growing body of biomedical literature is proving to be almost impossible. According to a study by Baasiri *et al*. (1) a researcher would have to scan 130 different journals and read 27 papers per day to follow a single disease, such as breast cancer. A more recent study by Lu (2) showed that the total number of references in MEDLINE, a central repository for scientific articles in the biomedical domain, now exceeds 25 million and is growing at more than 4% each year. It is also evident that a considerable amount of useful biological or biomedical knowledge is essentially buried in the form of free text, waiting to be found and transformed into more accessible formats. Swanson referred to such phenomena as ‘undiscovered public knowledge’ (3). The enormous challenges associated with keeping up or digging through this undiscovered public knowledge, especially in the area of biomedical knowledge, has led to the development of a number of text-mining tools aimed at supporting biomedical text extraction, fact finding and text summarization. Some of the better-known or more widely used tools include EBIMed (4), CiteXplore (5) and GoPubMed (6). Their intent has been to help life science researchers keep pace with the exploding quantity of scientific literature and to facilitate the discovery or re-discovery of important facts or unexpected associations. The latter task of ‘association discovery’ is of particular interest and is typified by queries such as ‘Find all genes that are associated with given disease’ or ‘Find all drugs that target a specific protein’ or ‘Find all toxins that damage a specific tissue’. These are queries that are either not easily performed or impossible to perform through a regular PubMed search. To address this task of association discovery we developed a relationship or association mining tool called PolySearch (7) (available online at http://wishart.biology.ualberta.ca/polysearch/). PolySearch was one of the first web-enabled text-mining tools to support comprehensive and associative text searches of PubMed abstracts. Specifically the original version of PolySearch supports ‘Given X, find all associated Y's’ types of queries, where X and Y are biomedical terms pertaining to human health and biology. X's can be genes, SNPs, proteins, diseases, drugs, metabolites, pathways, tissues, organs, and sub-cellular organelles or structures, or a general text keyword; while Y's can be any or all types mentioned above. PolySearch's search strategy is based on a critical assumption that the greater the frequency with which X and Y association occurs within a collection of sentences or database records, the more significant the association is likely to be. For example, if *Bisphenol A* (BPA) is mentioned 615 times in PubMed as being associated with breast cancer, and only 8 times being associated with colon cancer, then one is more likely to have higher confidence in the potential BPA-breast cancer association over the BPA-colon cancer association.

PolySearch has proven to be both popular and effective with >20 000 users and >150 citations. It has also served as an important text-mining and annotation system for the curation of a number of metabolomics databases including DrugBank (8), Human Metabolome Database (HMDB) (9), T3DB (10), YMDB (11), and ECMDB (12). PolySearch has also been used to assist in disease–gene discovery (13,14), protein–protein interaction studies (15,16), microarray data analysis (17), metabolome annotation (9,11,12,18), biomarker discovery (19), as well as in building and assessing other biomedical text-mining tools (20,21). PolySearch has also been featured in many published biomedical text-mining surveys and tutorials (2,13,22). However, a key limitation with PolySearch has been the long search times (2–3 min), its limited synonym set (thesauri) and its relatively small number of searchable databases. Indeed, since its introduction in 2008 many other searchable databases and electronic free-text collections have become available and many technological improvements in web interface design, text searching and text mining have taken place. Likewise, many PolySearch users have requested more search options such as MeSH terms, adverse health effects, animal taxonomies, medical terms, Gene Ontology and chemical ontology terms. In response to these requests and many ongoing technical developments we have created a second, much improved version of PolySearch (called PolySearch2). This faster (up to 25X) and much improved version now has a far more robust underlying framework. It also includes a much larger collection of databases (20 versus 7), search terms pairs (308 versus 66), thesauri (20 versus 9), terms (1 131 328 versus 57 706) and synonyms (2 848 936 versus 353 862) as well as a substantially improved and modernized interface and its underlying search algorithms. We have also upgraded the physical server to further improve its performance. A complete description of the new, updated PolySearch2 server follows.

### Improvements and enhancements in PolySearch2

PolySearch2 (http://polysearch.ca) features a number of improvements and enhancements including: (i) algorithmic improvements, (ii) an improved graphical interface and modernized web technology implementation, (iii) significant database and text search enhancements (iv) substantially expanded synonym sets and thesaurus types, and (v) improved caching and updating. These changes have also led to substantial performance improvements relative to the earlier version of PolySearch. Details regarding these changes and improvements are described below.

### Algorithmic improvements

PolySearch2 incorporates a number of algorithmic improvements aimed at strengthening the scoring, ranking and selection of association term candidates. These include: (i) a new ‘tightness measure’ to further discriminate association patterns, (ii) a ‘weight boost’ for database records to favour explicit database associations over free-text articles, (iii) a larger collection of system filter words and (iv) a filter to remove borderline associations.

PolySearch2 now uses a ‘tightness measure’ to reward more proximal word co-occurrences and penalize more distant word co-occurrences. Just as in the original version, PolySearch2 assigns relevant sentences into four categories (R1 [best], R2, R3 and R4 [worst]) based on a relevancy score as derived from the search query and the matched co-occurrence patterns. However, PolySearch2 now measures the word span between matched co-occurrence patterns found in a relevant sentence. In particular, it assigns higher relevancy scores to tighter patterns with fewer words separating the query term and target term(s), and lower relevancy scores to more relaxed patterns with a larger word span between the query term and the target term(s). An example R1 sentence with a tight co-occurrence pattern could be ‘Exposure to bisphenol A (BPA) increases the risk of breast neoplasms’, while an example R1 sentence with relaxed co-occurrence pattern could be ‘Bisphenol A may play a role in gene regulation pathways that are potentially related to the onset and development of breast cancer’. We found this tightness measure improves the scoring of co-occurrences and enhances PolySearch2's ability to distinguish genuine associations from incidental co-occurrences that arise by chance.

Unlike the original version of PolySearch, PolySearch2 now assigns greater weight to relevant database records than free-text articles. It has been previously shown (7) that including database records in the search process consistently improves association accuracy. Generally, database records contain high quality, well-structured and carefully curated knowledge whereas free-text articles generally contain more ambiguous, implicit knowledge. Therefore, it stands to reason that database records should be assigned higher credibility than text articles. However, given the shear volume of biomedical publications and the relatively small number of high-quality biomedical databases, one is more likely to find relevant free-text articles than database records. To counter this bias, PolySearch2 applies an empirically determined ‘weight boost’ to the information it finds in database records and assigns greater relevancy scores to relevant database records than free-text articles. The ‘weight boost’ reflects the difference in credibility associated with database records compared to sentences in free-text articles.

PolySearch2 also incorporates a more extensive collection of ‘system filter’ words than the original version of the program (29 718 filter words versus 7011 filter words). In particular, PolySearch2 now recognizes co-occurrence patterns more consistently thanks to this larger, more extensive collection of filter words. System filter words are essentially words that signify a strong association. For example, the word ‘catalyzes’ in ‘Enzyme X catalyzes reaction Y’ indicates a strong association between Enzyme X and reaction Y. The new and improved set of filter words were initially mined from the entire collection of MEDLINE abstracts using Natural Language Processing techniques. In creating PolySearch2's list of system filter words, we tagged the occurrence of all biomedical entities in the current collection of MEDLINE abstracts, extracted text flanking each pair of co-occurrence entities and classified the flanking text according to the co-occurring entity types. We then built N-gram models for common verbs, adjectives, adverbs and phrases present in the flanking text for each pair of co-occurrence entity types. The list was carefully assessed and manually curated to produce the final filter word set. This collection of system filter words helps PolySearch2 recognize strong associations from mere co-occurrences. It also allows it to perform consistently better at recognizing term associations than the original version of PolySearch.

The final algorithmic enhancement to PolySearch2 involved the application of a more stringent cut-off to boost precision at the cost of sacrificing a small degree of recall (i.e. the precision-recall trade-off). Associations discovered in PolySearch2 are ranked and sorted using Z-scores calculated from PolySearch2's raw relevancy score (see (7)). Associations with average relevancy scores are assigned zero Z-scores, as they represent borderline or marginal associations derived from a particular search. PolySearch2 now removes associations with zero Z-scores to boost its precision. This is done at the risk of removing a small number of possible genuine associations. For users concerned about the emphasis of recall over precision in their results, PolySearch2 also provides an option to include borderline cases (or 'zero Z-score' associations).

### Improved graphical interface and web implementation

**Figure 1.**
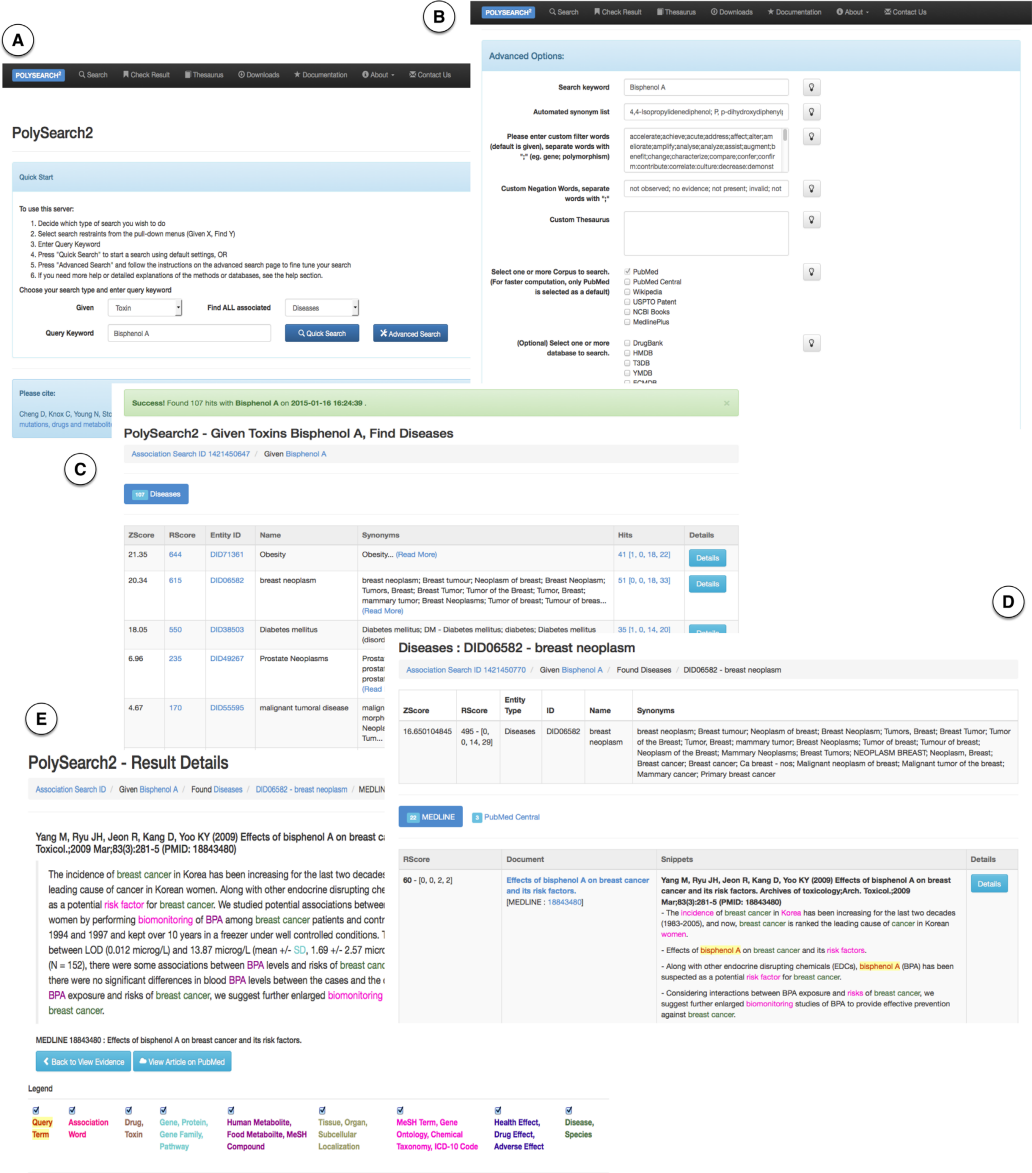
A screenshot montage of PolySearch2's query interface and result display showing (**A**) the PolySearch2 query submission form, (**B**) the advanced option page for further query refinement, (**C**) the PolySearch2 result overview table and (**D**) the detailed result page showing the supporting evidence for a single association.

PolySearch2 (http://polysearch.ca) features a completely re-designed web interface. Figure 1 shows a screenshot montage of various pages from PolySearch2's new web interface. Figure 1A shows the query submission page where users can initialize a search query. As with the original PolySearch, PolySearch2 still supports a ‘Given X, find all associated Y's’ type of query. Users can initialize a search by selecting the desired type of X (query term) and Y (target term) from pull-down menus and enter a search query keyword. At this point, user can submit a ‘Quick Search’ request or further configure the search using ‘Advanced Options’ (Figure 1B). Both of these features are new to PolySearch2. The Quick Search option will direct PolySearch2 to search previously computed cache results or to mine associations from the top 2000 relevant articles or database records across all text collections and databases. In the Quick Search, PolySearch2 automatically generates a synonym list (from the PolySearch2 thesauri) and proceeds with its regular searching, sorting, scoring, annotation and display (described in detail in (7)). ‘Advanced Options’ (Figure 1B) offers a greater degree of customizability to the search. For instance, users can edit the automatically generated synonym list (from the PolySearch2 thesaurus), edit custom filter words for identifying association patterns, provide custom negation words for filtering out sentences with negative associations, provide custom target terms to search, select or de-select source text collections and databases, indicate the number of documents to search, permit the inclusion or exclusion of hits with zero Z-scores (for higher recall) and/or provide an E-mail address for notifications.

Once a search is completed, the user will be redirected to a result overview page (Figure 1C) showing the associated entities of the selected target category (or all categories if the search is against ALL target categories). In Figure 1C, a screenshot listing the diseases found to be associated with the toxin BPA is shown. The resulting overview table is sorted by Z-scores in descending order, and can be sorted according to values in a certain column by clicking on the column header. The overview table lists the Z-score and PolySearch Relevancy Score (R-score) as well as the name and synonyms for each associated entity. Users can review query settings, browse through full tables in a printable format or download their results in JSON format by clicking the appropriate links on this page. Clicking on the ‘Details’ button on each row takes users to a detailed result page (Figure 1D) showing the supporting evidence in colour-coded and hyperlinked sentences from each relevant article in each text collection or biomedical database. For results with MEDLINE abstracts or PubMed Central articles, there is an additional ‘Details’ button for each row. Clicking on this specific ‘Details’ button takes user to view the full MEDLINE abstract in highlighted and hyperlinked text (Figure 1E). A result navigation bar with light grey background just below the headers of all result pages (Figure 1C–E) is provided for users to quickly review and navigate within the result hierarchy. These features are described in more detail on PolySearch2's Documentation web page.

**Figure 2.**
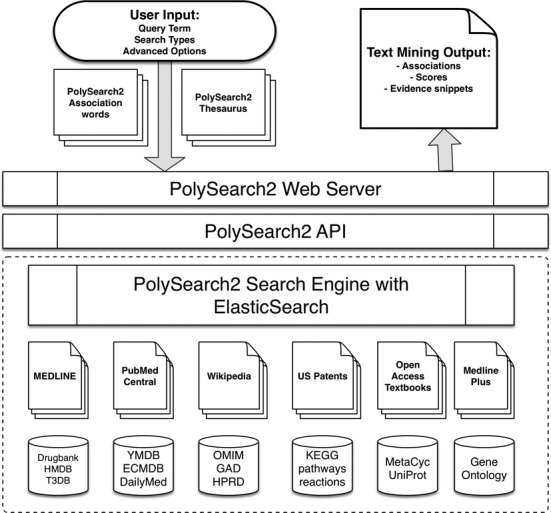
PolySearch2's system overview showing the architecture of PolySearch2 web server, API and the underlying search engine.

In addition to the substantially modified and updated graphical interface, PolySearch2 also underwent a complete upgrade and re-implementation of the web front-end using the latest web technology standards (HTML5 & Twitter Bootstrap). We have also upgraded the underlying physical server to further improve its performance. PolySearch2's back-end API and front-end web server are deployed on a dedicated tower server machine with eight cores operating at 1.4 GHz and multiple Solid-State Drives to facilitate rapid document retrieval and analysis. A PolySearch2 API for bulk text mining is also available upon request (with certain limitations). The architecture of PolySearch2 (see Figure 2) also allows it to be easily scaled to work across multiple machines on a computer cluster. It can also be adapted to a multi-computer cloud platform. PolySearch2 has been tested on a variety of platforms and is compatible with most common modern browsers (FireFox, Safari, Internet Explorer and Chrome) on both computer workstations and mobile devices. PolySearch2's analytical algorithm was implemented in Python and it uses ElasticSearch (see below) to manage document repository and cache results.

### Database and text search enhancements

For PolySearch2 we completely re-implemented the underlying text-mining framework based on the latest search engine technology (ElasticSearch, http://www.elasticsearch.org/) (see Figure 2). The utilization of ElasticSearch allowed us to internally host all text collections and databases (totalling 165 Gigabytes) across an ElasticSearch cluster running multiple nodes, and efficiently retrieve relevant documents. This has led to the ability to search against all thesaurus types simultaneously leading to a significant performance improvement and a nearly 25X acceleration in search times.

In PolySearch2, we have significantly expanded the number of text collections and databases (by more than 80%) to include a total of 6 free-text collections and 14 popular, text-rich bioinformatics databases. The latest release of PolySearch2 searches against over 43 million articles covering MEDLINE abstracts, PubMed Central full-text articles, Wikipedia full-text articles, US Patent abstracts, open access textbooks from NCBI and MedlinePlus articles. We believe these free-text collections cover a wide range of human knowledge from general information (Wikipedia, textbooks and MedlinePlus), to more specific biomedical knowledge (MEDLINE and PubMed Central), to technical innovations (US Patent applications).

While free-text collections represent a body of implicit knowledge, biomedical databases represent more specific or more quantitative, high quality curated knowledge. As illustrated in the original PolySearch paper (7), incorporating relevant database records into the search greatly enhances the resulting accuracy. To further improve on the performance of PolySearch2, we incorporated DrugBank (a popular drug and drug metabolite database) (8), HMDB (a human metabolite database) (9), T3DB (a toxin and toxin-target database) (10), YMDB (a yeast metabolome database) (11), ECMDB (an *Escherichia coli* metabolome database) (12), OMIM (Online Mendelian Inheritance in Man) (23), the UniProt database (24), the Human Protein Reference Database (25), DailyMed (FDA-approved drug listing information database) (26), KEGG reactions and pathways (27) and the MetaCyc (28) metabolic pathway database. For more information on PolySearch2's text collections and databases sources, please consult PolySearch2's Documentation web pages.

### Improved synonym collections

PolySearch2's custom thesauri or synonym collections are critical for the detection of biomedical terms mentioned in its databases and text collections. The original version of PolySearch had a thesaurus that consisted of nine categories with 57 706 terms, including names and/or synonyms for genes/proteins, gene families, diseases, drugs, metabolites, pathways, tissues, organs and sub-cellular organelles or structures. In PolySearch2, we have significantly expanded the number of thesauri from 9 to 20 categories, and from just 57 706 terms to over 1.13 million term entries with more than 2.84 million synonyms. PolySearch2's thesaurus collection now includes terms and synonyms for toxins (10), food metabolites, biological taxonomies (26), Gene Ontology terms (29), MeSH terms and MeSH compounds (30), along with ICD-10 (International Classification of Disease) medical codes (31).

PolySearch2's gene/protein thesaurus and gene family thesaurus were compiled from the latest release of UniProt (24), Entrez Gene (26), the Human Genome Organisation Gene Nomenclature Committee (32) and the Human Protein Reference Database (HPRD) (25). The disease thesaurus was compiled from Online Mendelian Inheritance in Man (OMIM) and the Unified Medical Language System (UMLS) (33). PolySearch2's drug and metabolite thesauri were compiled from the latest version of DrugBank (5) and HMDB (6), respectively. PolySearch2's pathway thesaurus was derived from names used for KEGG pathways (27) while PolySearch2's tissue thesaurus and organ thesaurus were created manually and the sub-cellular localization thesaurus was derived from the HPRD (25). PolySearch2's toxin thesaurus and food metabolite thesaurus were compiled from the latest version of the Toxic Exposome Database (T3DB) (10), and FooDB (http://foodb.ca/) respectively. The biological taxonomy thesaurus was derived from NCBI's taxonomy archive (26). PolySearch2's thesauri also feature many manually curated terms and synonyms for positive health effects, adverse health effects, drug actions, drug effects and chemical taxonomies. All of these thesauri may be searched via PolySearch2's Thesaurus page, and all may be downloaded via PolySearch2's Download page.

### Caching and auto-updating

PolySearch2 now has significantly expanded support for caching and automated update capability. Caching allows PolySearch2 to archive the results of common queries made by users so that if the same query is made by another user, then only a trivial update (if any) needs to be performed over the previously cached material. This leads to nearly instant (1–2 s) results for many common associative queries. PolySearch2 also regularly queries itself with thesaurus terms to increase its cache coverage far beyond what users may commonly generate.

The original version of PolySearch accessed the content of all (or nearly all) of its databases via the web. This ensured absolute data currency for all its databases, but it slowed the operation down substantially as all queries were subject to problems due to heavy website traffic loads, intermittent internet outages, varying data download speeds and the extra time needed to download large datasets over the web. Because PolySearch2 searches locally maintained databases on a (very large) local disk, none of these download or web access issues are encountered. However, moving to local databases meant that the data currency problem had to be addressed. Consequently, a number of custom scripts and ‘Cron’ jobs were developed so that new documents and new database updates are automatically retrieved on a daily basis and indexed to ensure that PolySearch2's text collections always contain the documents or data that are no more than 24 h old.

### Performance evaluation (PolySearch versus PolySearch2)

To assess the performance of PolySearch2, we conducted a speed test comparing only the speed of the original PolySearch with PolySearch2 on various queries with equivalent parameters. We then performed four evaluations to compare their accuracy. Finally, three additional evaluations were conducted to assess the performance of PolySearch2 on several novel search tasks. Performance statistics including precision, recall, f-measure and accuracy are presented in Table 1 for all seven evaluations. All seven evaluation datasets are available on PolySearch2's ‘Download’ page. Table 1 also lists some of the key feature differences between PolySearch and PolySearch2. We also evaluated PolySearch2's performance using the BioASQ (34) Task 3B semantic indexing and question answering gold standard training dataset and the results will be available on the ‘Evaluation’ page on the PolySearch2 website by May 1st, 2015.

**Table 1.** Performance evaluation and feature comparison of PolySearch2 versus PolySearch

	PolySearch				PolySearch2			
Prediction accuracy	P	R	F	Accu.	P	R	F	Accu.
#1 Disease/gene	0.6533	1.0000	0.7903	0.6533	0.8708	0.9091	0.8895	0.8525
#2 Drug/gene	0.7490	1.0000	0.8565	0.7490	0.9701	0.8351	0.8975	0.8571
#3 Protein/protein	0.8396	1.0000	0.9128	0.8396	0.9432	0.9326	0.9379	0.8962
#4 Metabolite/gene	0.7834	1.0000	0.8785	0.7834	0.9579	0.8619	0.9074	0.8614
#5 Drug/adverse effects	-	-	-	-	0.9233	0.8022	0.8585	0.7737
#6 Toxin/disease	-	-	-	-	0.9054	0.7864	0.8417	0.7810
#7 Toxin/adverse effects	-	-	-	-	0.8808	0.6822	0.7689	0.7854
Thesaurus categories	Nine categories				20 categories			
Thesaurus terms	57 706 terms with 353 862 synonyms				1 131 328 terms with 2 848 936 synonyms			
Filter words	7011				29 718			
Database numbers	One free-text collection and six databases				Six free-text collections and 14 databases			
Number of search types	66 query combinations				308 query combinations			
Analysis speed	6.5 documents per second				165 documents per second			
Mobile friendly?	No				Yes			

P stands for Precision, R stands for Recall, F stands for F-measure, and Accu. stands for accuracy. All evaluation datasets are available on PolySearch2's download page. Evaluation #1 assesses PolySearch2's ability to identify disease–gene association. Evaluation #2 assesses PolySearch2's ability to identify drug–gene/protein associations. Evaluation #3 assesses PolySearch2's ability to identify protein–protein interactions. Evaluation #4 assesses PolySearch2's metabolite–gene associations. Evaluation #5 assesses PolySearch2's ability to identify drugs with significant adverse effects, or ‘dangerous drugs’. Evaluation #6 assesses PolySearch2's ability to identify toxin–disease association. Finally, evaluation #7 evaluates PolySearch2's ability to identify toxin–adverse effect associations. Analysis speed is calculated based on multiple runs on a query with 10 000 relevant documents.

In the speed test, we calculated the speedup factor by dividing the execution time of the old PolySearch by the execution time of PolySearch2 on an identical set of 10 search queries. Both systems were located in the same network and both were accessed over the Internet. The cache look-up was disabled on both systems. The evaluation was carried out with 10 arbitrary keywords having more than 10 000 potentially relevant documents. The keywords were ‘Autism, Acetaminophen, Influenza, Rheumatoid Arthritis, *Escherichia coli*, Vitamin, Nucleus, p53, ATP, cancer’. A typical PolySearch2 query with 2000 or fewer relevant documents was completed in less than 20 s. On the other hand, a typical PolySearch query was completed in 2–5 min. We found that the time that both PolySearch and PolySearch2 take for keywords and search types is quite consistent, so document size is actually the main factor in determining overall execution time. Based on our data, PolySearch2 achieved a 5x to 25x speedup over PoySearch, depending on the number of documents (from 500 to 10 000) it analysed. In general, the more documents that are analysed, the greater the speedup, as PolySearch2's initialization overhead is amortized across a larger number of document analyses.

Next we evaluated PolySearch2's performance on four gold standard datasets (Table 1, tests 1–4). Specifically we evaluated PolySearch2's performance in mining: (1) disease–gene associations, (2) drug–gene associations, (3) protein–protein interactions and (4) metabolite–gene associations. PolySearch2's f-measures in these tasks were 88.95, 89.75, 93.79 and 90.74, respectively. Compared to the original PolySearch system, PolySearch2 achieved a 3–12% improvement in its association accuracy.

Finally, we evaluated PolySearch2's performance on three new gold standard datasets (Table 1, test 5–7). These tests were designed to identify (5) adverse drug effect associations for identifying 'dangerous drugs', (6) toxin–disease associations and (7) toxin–adverse effect associations. Performance statistics for the legacy PolySearch are not available for these datasets due to the novel search types and the size of the testing dataset. PolySearch2's f-measures on these tests were 85.85, 84.17 and 76.89, respectively.

The above result shows that PolySearch2 is substantially faster, more efficient and somewhat more accurate than the original PolySearch system. The improvement in computational efficiency is primarily due to the fact that we internally host all text collections and databases in PolySearch2. In the original PolySearch all queries were conducted through web-based APIs (which required querying and downloading abstracts from NCBI) or screen scraping on-line databases which is inherently slow. The automated update function in PolySearch2 helps ensure the currency of our corpus. The improvement in association accuracy can be attributed to the tightness measure we introduced to further discriminate matched association patterns, the assignment of weight boosting to database records in contrast to text articles and the imposition of more stringent cut-offs to boost precision at the expense of recall (precision-recall trade-off).

### Limitations

No text-mining system is perfect and certainly PolySearch2 is not without some limitations. One notable limitation is its inability to progressively or interactively adapt to specific search needs. High-end search engines such as Google and Yahoo monitor user feedback through surreptitious monitoring of user mouse clicks, web-page access and web-page dwell times. This helps these search engines customize or adapt to user preferences and needs. Ideally, PolySearch2 should be able to adapt to a search task by considering user feedback on the quality of discovered associations. For example, users may indicate certain associations to be false positives and in subsequent runs PolySearch2 should ideally learn from these negative examples and adapt itself to match a user's specific search needs and thereby achieve higher accuracy. We are currently testing several feedback systems and considering adding a ‘search satisfaction’ feedback system in future versions of PolySearch2. Another limitation with PolySearch2 (and for most text-mining systems) is its inability to self-assess its results and to extract specific knowledge on its own. While PolySearch2 performs well at extracting strong associations between biomedical entities it is not yet capable of assessing its discovered associations or extracted relations. For example PolySearch2 is able to identify a potential association between BPA and breast cancer but it is not able to infer a cause-and-effect relationship from the discovered association. Part of this limitation is due to the lack of training data to perform assessments and to extract relationships. To address this issue, we are hoping to use Machine learning (ML) and Natural Language Processing (NLP) techniques to eventually convert PolySearch2 from a simple association discovery tool to a more general knowledge extraction tool. We are currently working to incorporate this capability into future releases of PolySearch2.

## CONCLUSION

In this report we have described PolySearch2 (http://polysearch.ca), a web server designed to facilitate data mining and the semi-automated discovery of text associations between a wide range of biomedical entities. PolySearch2 supports ‘Given X, find all associated Ys’ type of queries with X and Y from more than 20 types of biomedical subject areas including human diseases, genes, SNPs, proteins, drugs, metabolites, toxins, metabolic pathways, organs, tissues, subcellular organelles, positive health effects, negative health effects, drug actions, Gene Ontology terms, MeSH terms, ICD-10 medical codes, biological taxonomies and chemical taxonomies. Some of the most significant improvements for PolySearch2 include a significant modernization of its underlying text-mining framework; a complete upgrade and re-implementation of the web interface using the latest web technology standards; a substantially improved algorithm for improved scoring and ranking of associations; significantly expanded custom thesauri and term collections; an expanded number of text collections and databases (by >80%); along with significantly improved support for caching and automated updating. PolySearch2 now offers greater speed (up to 25X faster), accuracy (3–12% improvement on f-measures), customizability (additional configurable options) and usability (modern and mobile-friendly web interface) than the original version. We believe that with these recent enhancements, PolySearch2 can better facilitate text-based discovery (and re-discovery) of latent associations among many types of biomedical entities and topics.
